# Programmed axon death: a promising target for treating retinal and optic nerve disorders

**DOI:** 10.1038/s41433-024-03025-0

**Published:** 2024-03-27

**Authors:** Andrea Loreto, Elisa Merlini, Michael P. Coleman

**Affiliations:** 1 John van Geest Centre for Brain Repair, Department of Clinical Neurosciences, University of Cambridge, Forvie Site, Robinson Way, Cambridge, UK; 2 School of Medical Sciences and Save Sight Institute, Charles Perkins Centre, Faculty of Medicine and Health, The University of Sydney, Sydney, NSW Australia

**Keywords:** Optic nerve diseases, Retinal diseases, Diseases of the nervous system

## Abstract

Programmed axon death is a druggable pathway of axon degeneration that has garnered considerable interest from pharmaceutical companies as a promising therapeutic target for various neurodegenerative disorders. In this review, we highlight mechanisms through which this pathway is activated in the retina and optic nerve, and discuss its potential significance for developing therapies for eye disorders and beyond. At the core of programmed axon death are two enzymes, NMNAT2 and SARM1, with pivotal roles in NAD metabolism. Extensive preclinical data in disease models consistently demonstrate remarkable, and in some instances, complete and enduring neuroprotection when this mechanism is targeted. Findings from animal studies are now being substantiated by genetic human data, propelling the field rapidly toward clinical translation. As we approach the clinical phase, the selection of suitable disorders for initial clinical trials targeting programmed axon death becomes crucial for their success. We delve into the multifaceted roles of programmed axon death and NAD metabolism in retinal and optic nerve disorders. We discuss the role of SARM1 beyond axon degeneration, including its potential involvement in neuronal soma death and photoreceptor degeneration. We also discuss genetic human data and environmental triggers of programmed axon death. Lastly, we touch upon potential therapeutic approaches targeting NMNATs and SARM1, as well as the nicotinamide trials for glaucoma. The extensive literature linking programmed axon death to eye disorders, along with the eye’s suitability for drug delivery and visual assessments, makes retinal and optic nerve disorders strong contenders for early clinical trials targeting programmed axon death.

## Abstract (zh)

程序性轴突死亡是通过药物预防轴突变性的一种有效途径, 是各种神经退行性疾病治疗有前景的治疗靶点, 引起了制药公司的极大兴趣。在这篇综述中, 我们着重介绍了这一用药途径在视网膜和视神经中激活的机制, 讨论了对开发眼部疾病及其他疾病治疗方法的潜在意义。程序性轴突死亡的核心通过两种酶, NMNAT2和SARM1, 在NAD代谢中起着关键作用。在疾病模型中大量临床前数据一致表明, 当这种机制成为治疗靶点时, 具有显著的、并且在某些情况下完全和持久的神经保护作用。动物研究发现的结果目前也在被人类基因数据所证实, 推动该领域迅速走向临床转化。随着临床转化的推进, 为针对其程序性轴突死亡的初步临床试验选择合适的疾病对其成功至关重要。我们深入研究了程序性轴突死亡和NAD代谢在视网膜和视神经疾病中的多方面作用, 讨论了严重急性呼吸系统综合征M1在轴突变性之外的作用, 包括其在神经元胞体死亡和光感受器变性中的潜在作用。文章同时讨论了人类遗传数据和程序性轴突死亡的环境触发因素。最后, 也讨论了针对NMNAT和SARM1的潜在治疗方法, 以及针对青光眼的烟酰胺试验。程序性轴突死亡与眼部疾病相关的大量文献, 以及眼对药物输送和视觉评估的适用性, 使视网膜和视神经疾病成为针对程序性轴突死的早期临床试验的重要适用者。

## Wallerian degeneration and programmed axon death

This review focuses on programmed axon death, and related mechanisms, in neurons of the human eye, but the thread underlying our current knowledge starts long ago in the tongue of the frog. Augustus Waller transected nerves there and observed ‘coagulation and curdling’ distal to the lesion site [1]. He predicted this process, which was subsequently named *Wallerian degeneration*, would be important for neurodegenerative diseases. Over the subsequent decades, there were reports of morphologically similar changes taking place in nerves in disease [2, 3] but there was no way to test for a similar mechanism. This changed abruptly in 1989 with the discovery by Hugh Perry and colleagues of the Wallerian degeneration slow (Wld^S^) mice. In these mice, a dominantly inherited, neuroprotective mutation enables tenfold longer survival of axons distal to an injury site [4–8].

**Fig. 1:**
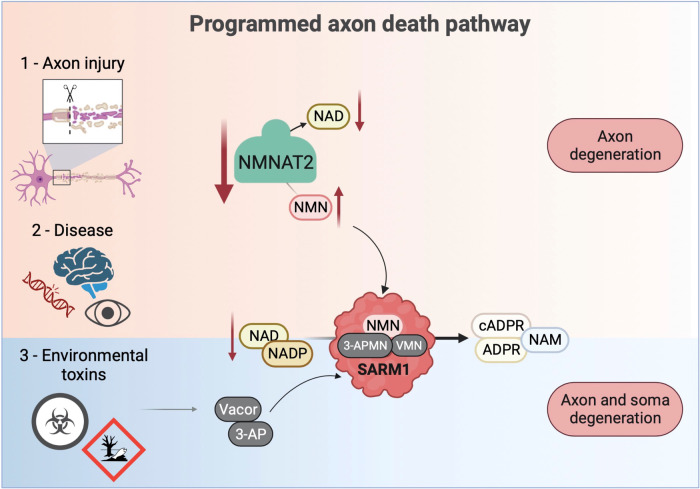
Programmed axon death pathway and triggers. Programmed axon death is triggered by various insults, including axotomy, neurodegenerative diseases, and exposure to environmental neurotoxins. These insults result in the depletion of NMNAT2 in the axon, a labile cytoplasmic enzyme that synthesises NAD from its precursor, NMN. NMNAT2 loss leads to an accumulation of NMN, which then binds to the pro-degenerative enzyme SARM1 and activates it. Once activated, SARM1 rapidly consumes NAD and causes axon degeneration. Notably, NMN analogues originating from environmental toxins, such as VMN and 3-APMN, can also bind and activate SARM1, bypassing the initial requirement for low NMNAT2 levels in the axon, triggering both soma and axon degeneration. This suggests that SARM1 toxicity is not restricted to axon-specific degeneration; instead, it can lead to the death of any cell that expresses sufficient levels of SARM1 and may lack compensation mechanisms. (NAM nicotinamide, 3-AP 3-acetylpyridine, NMN nicotinamide mononucleotide, VMN vacor mononucleotide, 3-APMN 3-acetylpyridine mononucleotide, NAD nicotinamide adenine dinucleotide, NADP nicotinamide adenine dinucleotide phosphate, ADPR adenosine diphosphate ribose, cADPR cyclic ADP-ribose, NAMPT nicotinamide phosphoribosyltransferase, NMNAT2 nicotinamide mononucleotide adenylyltransferase 2, SARM1 sterile alpha and TIR motif-containing protein 1).

Now that Wallerian degeneration could be delayed in mice, it became possible to test whether the same mutation delays or even prevents, axon degeneration in mouse models of disease where there is no physical injury. It quickly became clear that Wld^S^ could delay axon loss in many (but not all) disease models caused by gene mutation [9], toxins [10, 11], and metabolic defects [12–15]. This led to the term *programmed axon death*, which is the underlying mechanism shared by Wallerian degeneration after injury and these non-injury conditions (Fig. 1).

All of these early studies showed only a temporary delay of a few weeks or months in axon degeneration, and sometimes also in symptoms. However, increasing understanding of the molecular mechanism (see below) made it possible to activate the pathway very specifically, leading to striking findings of complete and permanent rescue of axons. There are at least two ways to do this: One is to remove a gene, *nicotinamide mononucleotide adenylyltransferase 2* (*Nmnat2)*, that is required to prevent programmed axon death from proceeding by default [16, 17]. The other is to directly activate the protein that executes the death programme, sterile alpha and TIR motif-containing protein 1 (SARM1), an enzyme that degrades nicotinamide adenine dinucleotide (NAD). SARM1 NADase is activated by the NAD precursor nicotinamide mononucleotide (NMN), but we found that it can also be activated even more potently, including in the retina, by a metabolite of the lethal environmental toxin, vacor [18]. This metabolite (vacor mononuclelotide, VMN) is an analogue of NMN and also activates SARM1 in, and kills, the neuronal soma. In each of these genetic and toxic models, axons and neurons are fully rescued by removing SARM1 (Fig. 1). The perinatal lethal phenotype of *Nmnat2* null mice, for example, is rescued for the entire two-year lifetime of laboratory mice. Importantly, both *NMNAT2* mutation and vacor cause disease in humans too [19–22], where we hypothesise that blocking SARM1 could be similarly effective.

## Programmed axon death in animal models of retinal and optic nerve disorders

**Fig. 2:**
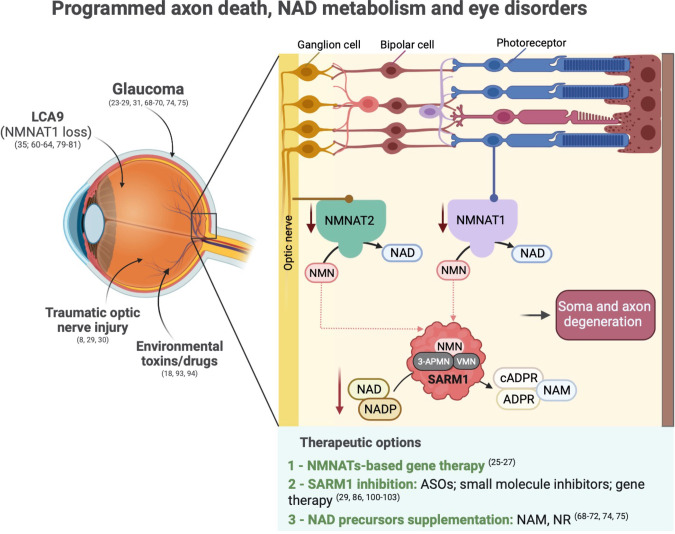
Programmed axon death, NAD metabolism and eye disorders. This figure highlights the potential involvement of programmed axon death in the pathogenesis of various retinal disorders, emphasising the roles of NMNATs and NAD homeostasis in maintaining retinal health. Programmed axon death can be activated in retinal cells through different mechanisms. Traumatic injuries and glaucoma may decrease NMNAT2 supply to the long axons of RGCs in the optic nerve, eventually leading to NMN accumulation and SARM1 activation, causing axon degeneration. In photoreceptor neurons lacking long axons, LoF mutations in NMNAT1, causing LCA9 in humans, have been associated with SARM1-dependent photoreceptor death. This suggests that, in the eye, SARM1 toxicity extends beyond the axonal compartment to affect neuronal soma as well. Toxins, such as vacor, 3-AP and vincristine, also cause ocular toxicity, which in this case is mediated by direct binding of their mononucleotide metabolites to SARM1, resulting in its activation. Programmed axon death is a preventable and druggable pathway, and various therapeutic options are under development to target eye disorders and other neurodegenerative diseases, as listed in this figure.

A key question for readers of this journal is whether programmed axon death occurs in retinal disorders. This too has been modelled in animals. Initial studies showed axon protection conferred by Wld^S^ following optic nerve crush, in a laser-induced model of glaucoma in rats, and spontaneously occurring glaucoma in DBA/2 J mice, where retinal ganglion cells (RGCs) were also rescued [8, 23, 24]. Subsequent research further expanded on these findings and highlighted the importance of NMNATs and NAD homeostasis in maintaining retinal health, suggesting a potential involvement of reduced NMNAT2 activity in the pathogenesis of glaucoma [25–28] (Fig. 2).

Several research groups have also showed that SARM1 drives degeneration of RGCs and their axons after traumatic optic nerve injury [29, 30], in a neuroinflammatory model of glaucoma [31], in the silicone oil-induced ocular hypertension [29], and after mitochondrial dysfunction [32] and excitotoxicity [33]. Remarkably, SARM1 also triggers photoreceptor death in genetic mouse models of human photoreceptor disorders [34, 35], and after toxic [36] insults (Fig. 2). Taken together, these studies suggest that various insults impacting different types of retinal cells ultimately lead to optic nerve and retinal degeneration through programmed axon death.

## Understanding the mechanism of programmed axon death

The key event that initiated understanding of programmed axon death at the molecular level was the identification of the Wld^S^ mouse, where injury-induced Wallerian degeneration was delayed from around 1.5 days after nerve injury to 2–3 weeks [4]. Mendelian inheritance of this phenotype meant the gene could be mapped to a specific chromosomal region and eventually identified [5, 7]. Structure-function analysis of the causative protein led to the conclusion that the protective mechanism involved a gain-of-function of NMNAT activity (the synthesis of NAD from its precursor NMN) within axons, that does not alter basal NAD levels but does stabilise both NAD and NMN after nerve injury [6]. This gain-of-function comes about in the Wld^S^ mouse through a genetic ‘accident’ that fuses the full coding sequence of NMNAT1, normally a nuclear enzyme, to a short region of ubiquitin ligase UBE4B. This has the effect of relocating some NMNAT1 into axons [37, 38]. Other ways of translocating NMNAT1 into axons had similar effects but when NMNAT1 remained nuclear there was no protection [37, 39, 40].

If a gain-of NMNAT activity in axons could delay Wallerian degeneration, it was pertinent to ask whether loss of the same activity could drive axon death through this same mechanism. This was tested by knockdown of all three isoforms, resulting in the discovery that removing NMNAT2 activates axon degeneration both in vitro and in vivo, which could be blocked by Wld^S^ in both cases [41, 42]. NMNAT2 is an unstable protein with a half-life in some cells as short as 40 min [43], whereas NMNAT1 and Wld^S^ are far more stable, so we proposed the model that axon injury, or impairment of NMNAT2 axonal transport, rapidly deplete axons of this essential activity through normal turnover that is no longer balanced by delivery of newly synthesised protein [41] (Fig. 1). Considering the time taken for axonal transport to deliver proteins along their full length (up to several days for the longest axons), and the discovery of NMNAT2 mRNA in axons [44], the situation is undoubtedly more complex. For example, the portioning of NMNAT2 between a vesicular and cytosolic form influences its stability and potential to preserve axons considerably [43, 45]. However, the absolute requirement of NMNAT2 for vertebrate axon survival, unless programmed axon death is blocked in some other way, remains a good working model that also explains findings in *Drosophila* [46, 47] and human genetics [20–22].

Discovery of the role of SARM1 in axon survival came from *Drosophila* screens for other modifiers of programmed axon death. After showing that murine Wld^S^ could preserve injured axons in *Drosophila*, indicating functional conservation of the mechanism over considerable evolutionary distance [40], Marc Freeman and colleagues searched for an effector of the pathway using random chemical mutagenesis and extensive phenotypic screening in fruit flies. Multiple loss-of-function (LoF) alleles in the *Drosophila* orthologue of SARM1 (dSarm) were found to phenocopy the expression of Wld^S^, preserving injured axons very strongly, and SARM1 null mice were then found to have the same phenotype [48]. An RNAi project in murine neuron cultures independently identified the same protective effect of knocking down SARM1 shortly after [49].

We then found that removing SARM1 conferred lifelong rescue on the NMNAT2 null phenotype, but without completely stabilising either the substrate (NMN) or product (NAD) of NMNAT2, placing SARM1 activation downstream of NMNAT2 loss in the pathway [16, 17] (Figs. 1, 2). The key activity was identified by the Milbrandt and DiAntonio groups as an intrinsic NADase [50], or possibly a closely related activity at the same catalytic site [51], and the activation mechanism was shown to involve the build-up of NMNAT2 substrate NMN when NMNAT2 is lost from axons [52–54], which then activates SARM1 [55]. NAD competes with NMN for binding to the SARM1 regulatory domain and thus at high levels can be protective [51, 56, 57], while analogues of nicotinamide (NAM) such as vacor and 3-acetylpyridine (3-AP) are metabolised into analogues of NMN, which activate SARM1 causing substantial toxicity [18, 51, 58] (Fig. 1).

## Beyond axon degeneration: SARM1 activation kills neuronal soma

Programmed axon death has conventionally been associated with a specific axon degeneration pathway exclusive to the axon itself. Axons are notably susceptible to programmed axon death as neither nuclear NMNAT1 nor mitochondrial NMNAT3 can compensate for the loss of the labile and primary axonal isoform NMNAT2, whose levels in axons decrease following various toxic insults (Fig. 1). However, recent findings have unequivocally shown that activation of SARM1 leads to death of neuronal soma and, more broadly, cell death. There are at least two good examples of this: first, when SARM1 is directly activated or has higher activity. Two paradigms are the death of neuronal soma after direct activation of SARM1 by the neurotoxin vacor (Fig. 1) and the overexpression of SARM1 gain-of-function (GoF) variants, which are prevalent in amyotrophic lateral sclerosis (ALS) patients (see below) [18, 59]. The second instance is when the activity or levels of other NMNAT isoforms are compromised, especially in cells where alternative isoforms cannot compensate sufficiently, leading to NMN accumulation and SARM1 activation [55]. A very relevant example of this is the neurodegeneration of photoreceptors due to NMNAT1 loss. In this neuronal type, which lacks a long axon, NMNAT1 has been proposed to play a more significant, possibly extranuclear role, potentially compensating for low cytosolic levels of NMNAT2. As seen in axons after NMNAT2 depletion, loss of NMNAT1 results in SARM1-dependent death of photoreceptors [35] (Fig. 2). This holds clinical relevance as LoF mutations in NMNAT1 underlie Leber congenital amaurosis type 9 (LCA9) [60–64].

In essence, activation of SARM1 is not limited to axon-specific destruction; rather, it can cause death of any cell in the body that expresses sufficient levels of SARM1 and/or lack compensation mechanisms (Figs. 1, 2).

## Impaired NAD homeostasis in retinal degeneration: is this caused by programmed axon death activation?

The studies mentioned above show that targeting key enzymes that regulate programmed axon death protect from retinal degeneration and provide direct evidence of an involvement of this mechanism in the pathogenesis of retinal degeneration, at least in animal models. Yet, the role of programmed axon death in neurodegenerative diseases of the eye could be even broader. NMNATs and SARM1 are key regulators of NAD metabolism and there is growing evidence of the importance of NAD homeostasis for maintaining eye health. There are indications of NAD impairment in multiple eye disorders, and it may even decline with age [25, 65–67]. In a series of important studies, Williams and colleagues have shown that supplementation with the NAD precursor NAM, a form of vitamin B3, is remarkably neuroprotective in the DBA/2 J glaucoma model [25, 26, 68–70]. Also nicotinamide riboside (NR), another NAD precursor, is neuroprotective in animal models of RGC degeneration [71, 72]. Recent data also indicate that patients with primary open-angle glaucoma have reduced serum levels of NAM [73]. On the back of the positive results from animal studies, two clinical trials explored the benefits of NAM supplementation in glaucoma patients, yielding promising results [74, 75]. Currently, larger trials are in progress.

However, these studies are insufficient to conclusively demonstrate that the beneficial effects of NAM supplementation are mechanistically linked to the interference with programmed axon death, and the extent to which activation of programmed axon death contributes to impaired NAD homeostasis in retinal disorders. The complexity of NAD metabolism, the involvement of numerous enzymes that regulate NAD metabolism beyond the programmed axon death pathway, and the impact of NAD precursor supplementation on various intracellular pathways essential for cell survival [76] add layers of complexity. Looking ahead, a key goal in the field is to deepen our understanding of how NAD homeostasis is impaired in retinal disorders and the roles played by programmed axon death and other enzymes in NAD metabolism. For instance, it is important to determine whether SARM1 deficiency confers protection to RGCs and axons in the DBA/2 J glaucoma model, given the high effectiveness of NAM treatment in this context. Additionally, investigating whether NAM blocks SARM1 activation in the same model would offer stronger support for the notion that the neuroprotective mechanism of NAM is, at least to some extent, linked to interference with programmed axon death. Nevertheless, the clear outcome of these studies is that targeting NAD metabolism with vitamin supplements, NMNATs and blocking SARM1 all result in neuroprotection of different retinal cells and against diverse insults.

## Disease models: what they tell us and what they don’t

In addition to disorders of the retina and optic nerve, many other models of disease have been alleviated in mice, rats, zebrafish and cell culture by overexpressing NMNATs or removing or blocking SARM1 [14, 77]. While this is compelling evidence of a role for programmed axon death, and SARM1 activation by other mechanisms, in neurological and eye disorders, disease models have important limitations that mean direct extrapolation to the corresponding human disorder can be misleading. Not only do aspects of the genetics, anatomy and lifespan of the species used, and the environment in which they are housed, differ from those of humans, but differences in the way we induce disease in animal models are frequently underappreciated. Most human disease is multifactorial. Genetic and environmental heterogeneity across human populations mean different risk factors, even at modest levels, combine to cause similar outcomes in different patients. In contrast, most animal models involve hitting just one risk factor very hard on a genetically homogeneous background. Examples include the use of fully penetrant, and sometimes also overexpressed, gene mutations, high doses of toxins or a substantial rise in raised intraocular pressure (IOP) to model glaucoma when IOP is only one of the risk factors in humans [65]. Ageing is also often not represented in many animal models on cost and other practical grounds. Thus, to really understand the importance of programmed axon death in human disease, we need real-world data from human populations.

## Human genetic studies of programmed axon death

One type of real-world human data is of course a clinical trial. However, moving directly from animal data with such limitations as those noted above to clinical trials, with the associated ethical and cost concerns, is likely to be one important contributor to high failure rates. Very effective protection from one pathway such as programmed axon death may translate very well in specific patients where this pathway plays a large role, but the signal could be completely masked by ‘noise’ from patients where other pathways are the major drivers of disease. Information gained from human genome and exome sequencing offers three alternative ways to gain real-world human data and is the key to a much-needed personalised medicine approach.

The first is to identify mutations in human disease whose functional consequences closely resemble those in animal models, suggesting causation. There are several examples, all in rare disorders so far. Homozygous null *Nmnat2* mice die perinatally with axon growth and muscle development defects [42], while the equivalent condition seen in two human cases was even more severe with *in-utero* lethality and a complete absence of skeletal muscle [21]. *Nmnat2* hypomorphic mice have an early-onset sensory phenotype and reduced sensory axon numbers [78], and biallelic partial LoF in humans is also associated with sensory symptoms, varying degrees of motor impairment, and axon deficits in sural nerve [20, 22]. Also LoF mutations in *NMNAT1* were identified in patients with LCA9 [60–64]. Studies in rodents confirm the importance of NMNAT1 for photoreceptor cells survival and maintenance of a healthy retina [79–81]. Strikingly, *Sarm1* deletion rescues photoreceptors and retinal degeneration caused by *Nmnat1* deletion in mice, suggesting an involvement of SARM1 in the pathogenesis of LCA type 9 [35] (Fig. 2).

The second source of real-world human genetic data is gene variants that increase disease risk. This is seen with *SARM1* GoF alleles that are enriched in sporadic ALS patients relative to matched controls [59, 82]. These hyperactive alleles do not appear to be the sole cause of disease in these patients, with evidence of other, partially penetrant neurodegenerative mutations in several cases, but they are harmful to neurons and are likely to contribute to disease. It will be important to determine whether *SARM1* GoF contributes to other human neurological disorders, although not all fields have yet had the foresight, funding and co-ordination needed to make so much whole-genome sequence data publicly available as in ALS [83]. When this happens, it will be game-changing.

Third, when there are protective genetic variants in humans, in this case *SARM1* LoF and dominant negative alleles [84], it becomes possible in principle to use Mendelian randomisation as the basis of a ‘natural clinical trial’ [85], testing whether such variants lower disease risk. If they do, then SARM1-blocking drugs would seem likely to do the same. Evidence from animals confirms that loss of just a single *SARM1* allele protects axons from multiple stressors [86] so basing such a study on heterozygous carriers may be sufficient. This may require very large numbers of patients, well-matched controls and more LoF alleles than we currently know of, but it is certainly an important prospect for the future. In the meantime, the viability of humans with dominant negative *SARM1* mutations supports the likelihood that drugs blocking *SARM1* will be reasonably safe. The potential to study the impact of druggable risk factors in this way is a compelling reason to gather extensive whole-genome sequence datasets in common eye disorders.

## Environmental activators of programmed axon death

In addition to this genetic evidence, it is now clear that programmed axon death can be activated by at least three types of environmental risk factor. The first is injury. Transection injury is described above, but this also extends to traumatic brain injury models and to raised IOP in glaucoma models, which are alleviated by Wld^S^ or SARM1 deletion [23, 24, 26, 87–89]. The likely explanation is that high IOP disrupts axonal transport at the optic nerve head [90], limiting the supply of NMNAT2 to distal axons. The second environmental trigger is toxins. These can act by activating SARM1, as for example in vacor toxicity (see above), or by impairing the delivery of NMNAT2 by axonal transport, the likely mode of action in vincristine neuropathy [91, 92]. Both show ocular toxicity as well as damage to other types of neuron [18, 93, 94] (Fig. 2). Final, SARM1-dependent axon loss is caused by several viruses, including rabies and zika, with related phenomena also reported for West Nile virus [95–98]. As the eye is an important route of infection for a number of viruses and many cause optic neuropathies, including (but not limited to) herpes viruses, West Nile virus, Epstein-Barr virus [99], it will be important to determine whether viral activation of SARM1 plays a role in any retinal disorders.

## Therapies to block programmed axon death: the path towards translation

Programmed axon death is druggable and can be entirely prevented when specifically activated. Evidence from preclinical studies and initial human data indicate that multiple pathological processes converge on this pathway, potentially playing a role in more than one neurodegenerative disorder. The permanent rescue of axons observed in preclinical studies through SARM1 deletion, along with the overall health and normal lifespan of SARM1-deficient mice, has led to the focus of numerous drug development programs on inhibiting SARM1 activity. Numerous therapeutic approaches have demonstrated efficacy in animal and cellular studies, encompassing small molecule inhibitors, gene therapy, and antisense oligonucleotides (ASOs) targeting SARM1 [29, 86, 100–103]. While NMNATs show promise as therapeutic targets too, they have received somewhat less attention in drug development efforts, primarily due to the greater ease of inhibiting an activity rather than enhancing or maintaining one. Consequently, the focus has predominantly centred on SARM1. Nevertheless, NMNAT-based gene therapy has demonstrated significant potential for retinal degeneration in animal studies [25–27] and may be particularly well-suited for treating eye disorders, given the ease of translatability and the existence of FDA-approved retinal gene therapy [104]. Our understanding of programmed axon death and the mechanisms of its regulators is expanding continuously, raising optimism that an effective drug to block programmed axon death will become available in the coming years.

Regardless of the eventual choice of therapeutic approach, a key question arises: which disease should be prioritised for the first programmed axon death clinical trial? The rationale, supported by extensive preclinical data, strongly suggests that the most substantial effect sizes are likely to be observed in diseases resulting from mutations in NMNAT2 or SARM1. However, these are rare diseases, often characterized by variable and sometimes severe, early-onset, and widespread neurodegenerative phenotypes that vary depending on the specific mutation, and which may necessitate systemic inhibition of programmed axon death. Starting with a neurodegenerative eye disease offers several advantages. The eye is particularly amenable to gene therapy and ASO therapies [105] and there are relatively straightforward functional tests for measuring treatment outcomes. The importance of NAD metabolism and programmed axon death in retinal and optic nerve disorders is also well documented in the literature. Conducting a local treatment directly administered to the eye, as opposed to a systemic approach, which demonstrates both safety and efficacy of blocking programmed axon death in humans, could serve as a steppingstone towards extending clinical trials to other complex and multifactorial disorders.

Choosing which eye disorder to target first is not straightforward though. Patients with LCA9 caused by NMNAT1 mutations stand to benefit from a drug blocking programmed axon death. However, the early onset and severe phenotypes of this disorder necessitate intervention at pre- or early symptomatic stages and might limit the available patient pool. Furthermore, it is key to determine whether the therapeutic effects of NAM supplementation in glaucoma are, at least in part, due to the inhibition of programmed axon death. If this were the case, the already available data from NAM clinical trials would provide further human evidence of programmed axon death involvement in glaucoma. This would open the possibility of a combined approach, using both NAM supplementation and drugs targeting programmed axon death regulators, to potentially yield the most effective therapeutic outcomes. Larger clinical trials on NAM supplementation in glaucoma are currently underway, and in the coming years, we hope to gain a clearer understanding of this promising therapeutic avenue. Lastly, accumulating more data in human neurons via induced pluripotent stem cell (iPSC) models and human retinas from patients, while seeking markers of programmed axon death activation, will be essential for guiding disease and patient selection.

## Conclusions

In summary, SARM1 is a pro-degenerative enzyme whose activity is kept at a low, safe level by NMNAT2 within long axons, including optic nerve, and by NMNAT1 in the soma or neuron types without long axons such as photoreceptors. SARM1 becomes activated when the respective NMNAT is disrupted by mutation or (for NMNAT2) by axonal transport deficiency, underlying involvement of the pathway in LCA9 and glaucoma models respectively. Neurotoxins whose metabolic products mimic its natural activator, NMN, also activate SARM1, killing cells and axons, while some viruses also cause SARM1-dependent death. With multiple drug discovery efforts and NAD precursor-based therapies underway, eye research has crucial roles in effective testing and use. Protection is strongest when the pathway is activated most specifically, probably including LCA9, where NMNAT1 activity is lost. Genomic studies to identify other relevant diseases and patients will benefit from well-coordinated whole-genome sequencing (WGS) initiatives. Finally, the eye is an ideal site for clinical trials, with easy accessibility allowing easy delivery of drug candidates, visual assessment and biomarker sampling, and its relatively contained structure supporting safety. Therapies successfully developed in this way are likely to have further applications both in other eye disorders and in the wider nervous system.
